# Parents’ and Early Childhood Educators’ Perceptions after the Implementation of the Pragmatic Intervention Programme (PICP)

**DOI:** 10.1177/23969415251330465

**Published:** 2025-05-12

**Authors:** Tatiana Pereira, Ana Margarida Ramalho, Marisa Lousada

**Affiliations:** 1 RISE-Health, 56062 University of Aveiro, Aveiro, Portugal; 2 Center of Linguistics of the University of Lisbon (CLUL), Faculty of Letters, 37809 University of Lisbon, Lisboa, Portugal; 3 School of Health Sciences of Alcoitão, Alcabideche, Lisboa, Portugal; 4 School of Health Sciences (ESSUA), 56062 University of Aveiro, Aveiro, Portugal

**Keywords:** Autism spectrum disorder, developmental language disorder, parents, early childhood educators, pragmatic intervention programme, evidence-based practice

## Abstract

The presence of pragmatic language difficulties can be lifelong for children with autism spectrum disorder (ASD) and developmental language disorder (DLD). Implementing evidence-based practices integrates the best research evidence, the clinical expertise of the professionals delivering the intervention and the perspectives of parents/caregivers. Nevertheless, research on parents’ and early childhood educators’ perspectives on the perceived effectiveness of pragmatic language interventions is scarce. This study aims to analyse parents’ and early childhood educators’ perceptions after the implementation of the Pragmatic Intervention Programme (PICP) to preschool-age children with ASD and children with DLD. As a part of a broad research project, a survey was conducted using an adaptation of a satisfaction survey. Data from 72 participants among parents (*n* = 36) and early childhood educators (*n* = 36) were collected immediately after the implementation of the PICP. The survey includes 11 statements, individually scored between 1 (*totally disagree*) and 7 (*totally agree*). The average score obtained from the parents’ perspective about the intervention impact was 6.83 ± 0.29. For early childhood educators, the average score was 6.60 ± 0.49. The results indicate that parents and early childhood educators considered this intervention appropriate and effective for improving the pragmatic language skills of preschool-age children with ASD and DLD.

## Introduction

Pragmatics is defined as the appropriate use of language in social contexts to serve communicative purposes (Alduais et al., 2022). Children diagnosed with autism spectrum disorder (ASD) and developmental language disorder (DLD) often have pragmatic language difficulties^1^ that can negatively affect learning, socialisation, and mental health. These difficulties usually persist into adulthood (McGregor, 2020), affecting daily function and well-being (Papadopoulos, 2018), so timely interventions are crucial to minimise the long-term impact of pragmatic language difficulties on people's lives (Brinton & Fujiki, 2019; Rinaldi et al., 2021).

The literature reports both similarities and differences in the pragmatic language of children with ASD and children with DLD.

Andrés-Roqueta and Katsos (2020) found many similarities between the pragmatic abilities of children with ASD and children with DLD using linguistic-pragmatic tasks and social-pragmatic tasks. More specifically, their study showed that both children with ASD and children with DLD faced severe difficulties in the linguistic-pragmatic domain, and their performance in the task was similar. In contrast, in the social-pragmatic task, children with ASD performed worse than children with DLD due to their difficulties with the Theory of Mind.

Georgiou and Spanoudis (2021) analysed the language profile of children with ASD, children with DLD, and typically developing peers using the second edition of the Children's Communication Checklist (CCC-2) (Bishop, 2003) separated into two subscales: the general communication composite (GCC), which includes the subscales of speech, syntax, semantics, coherence, inappropriate initiation, stereotyped language, use of context and nonverbal communication, and the social interaction deviance composite (SIDC), which is obtained by summing the scales for inappropriate initiation, nonverbal communication, social relations, and interests and then subtracting the scales for speech, syntax, semantics, and coherence. The results showed that both clinical groups face pragmatic language difficulties, but children with DLD exhibited only in GCC, while children with ASD presented difficulties in both GCC and SIDC. In the study by Geurts and Embrechts (2008), by analysing the results of CCC-2, the authors found that children with ASD showed statistically significantly more difficulties in the use of context than children with DLD, while children with DLD presented significant differences compared to typically developing children. Additionally, children with ASD had difficulties with initiating conversation, nonverbal communication, social relationships, and interests compared to both children with DLD and typically developing peers, while children with DLD did not present any differences compared to typically developing children. Both children with ASD and DLD revealed difficulties in speech output, syntax, coherence, and semantics, as well as in stereotyped language, when compared to typically developing preschoolers.

Andreou et al. (2022) carried out a systematic review to analyse the literature on the pragmatic language of children with ASD and DLD. The authors included information about general and specific aspects of pragmatic language abilities to generate an accurate profile for both clinical groups. According to the authors, the term “general pragmatic language” concerns the fundamental features of pragmatics, evaluated through measures of global pragmatic function. Specific aspects of pragmatic language were identified as skills influencing pragmatic language abilities, including using fillers, narrative skills, employment of quantifiers, and article choice. The study revealed similarities and differences in the two clinical groups’ general and specific pragmatic abilities.

Overall, the literature describes similarities and differences and further research is needed to clarify the pragmatic language profile of children in the two clinical groups (Andreou et al., 2022). Although the extent of impairment in each population and the underlying causes are under debate (Andrés-Roqueta & Katsos, 2020), there seems to be a consensus about the severity of the difficulties between conditions, and studies indicate that children with ASD exhibited more severe pragmatic language difficulties than children with DLD (Andreou et al., 2022; Hage et al., 2021).

Over the past decades, there has been a growing emphasis on developing intervention programmes to promote the acquisition and to improve children's pragmatic language across neurodevelopmental disorders, including ASD and DLD (Jensen de López et al., 2022; Parsons et al., 2017). Nevertheless, for DLD, there are significantly fewer intervention studies aimed at pragmatics than in other language domains (Jensen de López et al., 2022). These programmes often involve collaboration between speech and language therapists (SLTs), early childhood educators, and caregivers to create supportive learning environments that facilitate the generalisation of skills beyond structured intervention sessions (Klatte et al., 2020). The efficacy of the existing studies varies, and considering the high diversity in reporting relevant components to confirm intervention effectiveness; it is difficult to give recommendations regarding the intensity and duration of a specific intervention (Jensen de López et al., 2022). Moreover, the long-term effects of these interventions and the generalisation of learning to new contexts are largely unknown. Outcome measurement often assesses pragmatic language in the context in which the intervention was administered or via a decontextualised assessment instrument, so conclusions cannot be drawn (Parsons et al., 2017).

For these two conditions, despite the similarities and differences described in the literature (Andreou et al., 2022), fewer studies have analysed and compared the practice patterns of the SLTs in the field of pragmatic language, considering DLD and ASD. A recent study was conducted in Portugal to analyse how these similarities and differences influence the clinical practice of Portuguese SLTs (Pereira et al., 2025a). Concerning intervention, differences were found in the intervention setting (the most frequent intervention setting reported for ASD was the clinic, and for DLD were the early childhood educational settings) and communicative partners involved (the most frequently mentioned communicative partners involved in the intervention process were caregivers when managing children with ASD, and early childhood educators when managing children with DLD). The similarities included type of intervention (mixed), frequency (weekly), and length of the sessions (30–45 min). Furthermore, over 90% of the SLTs did not follow any specific method, programme, or approach, regardless of the child's condition. This can be related to the fact that there is only one intervention programme developed and content validated for preschool-age European Portuguese children with pragmatic difficulties—Pragmatic Intervention Programme (PICP) (Pereira et al., 2019)—which was found to be used by SLTs (the minority who reported following a specific method, programme, or approach) to improve pragmatic language skills in both conditions (the majority of the SLTs who participate in the survey used informal intervention procedures). The authors emphasise that the similarities and differences were mainly due to the scarcity of research in clinical pragmatics, which were also reflected in the needs perceived by SLTs in this field and the degree of confidence with which they work with these children (Pereira et al., 2025a).

The PICP is a manualised intervention programme that aims to promote pragmatic language competencies among preschool-age children with difficulties in this language domain. The manual provides goals, activities, procedures, and strategies that can be applied to improve several pragmatic language competencies. Eleven competencies are part of this programme: (1) eye contact, (2) joint attention, (3) turn-taking, (4) communicative response, (5) communicative initiative, (6) communicative functions, (7) comprehension and expression in verbal and nonverbal communicative contexts, (8) cohesion, (9) inferential comprehension, (10) conversation, and (11) figurative language. Additional information regarding the development and content validation of the PICP can be consulted in Pereira et al. (2021). Following the principles of the PICP, this programme should be implemented by an SLT according to the child's needs, and the competencies addressed should be worked on with different communicative partners (e.g., peers, early childhood educators) and in multiple contexts (e.g., home, early childhood educational settings) to promote skills generalisation from the very beginning (Pereira et al., 2021).

The effectiveness of the PICP was studied through a non-randomised controlled trial. This quasi-experimental study was carried out in early childhood educational settings and included children with ASD (*n* = 20) and children with DLD (*n* = 16). Thirty-six children were assigned to the experimental (*n* = 22) or control group (waiting list) (*n* = 14). Each child attended 24 PICP-based intervention sessions provided by an SLT. The primary outcome measure was a Goal Attainment Scale (GAS) adapted from Adams and Gaile (2020), and rated by parents and early childhood educators after the intervention. The secondary outcomes were *Escala de Avaliação de Competências Comunicativas* (EAC) (Seabra et al., 2021) rated by parents and early childhood educators and *Teste de Linguagem—Avaliação da Linguagem Pré-Escolar* (TL-ALPE) (Mendes et al., 2014), applied by two SLTs blind to the aims of the study and group allocation. The results showed that all children achieved clinically significant progress in GAS after the treatment. Statistically significant differences (*p* < .05) were observed between groups in the EAC and TL-ALPE after the intervention of the experimental group. The interaction between time (before/after intervention), group (control/experimental), and condition (ASD/DLD) was only statistically significant for the EAC rated by parents. After the intervention of the control group, the post-intervention scores (EAC and TL-ALPE) of both groups were compared and no statistical differences (*p* < .05) were found. This means that regardless of group and condition, the children improved their language competencies similarly after the intervention. These findings suggest that PICP is an effective intervention programme for improving the pragmatic language skills of preschool-age children with ASD and children with DLD and also promotes other language skills, namely semantic and morphosyntactic (Pereira et al., 2025b). A full description of the trial methods and results is provided by Pereira et al. (2025b).

Considering the systematic reviews about pragmatic language interventions conducted by Parsons et al. (2017) involving children with ASD, and Jensen de López et al. (2022) including children with DLD, it is clarified that several effectiveness studies have been carried out, which are essential for parents and professionals to be able to make informed decisions based on the best external evidence of the intervention effects. Nevertheless, as a central part of evidence-based practice, the clinical expertise of the professionals delivering the interventions and the caregivers’ perspectives should also be considered (Law et al., 2015).

An improved understanding of the parents’ views, experiences, and preferences is crucial to inform intervention and policy and enhance outcomes (Jensen de López et al., 2021). Parents have in-depth knowledge about the child's social communication skills, which is necessary to plan detailed and personalised interventions (Baxendale et al., 2013). Thus, parents’ opinions, observations, understandings, and lived experiences concerning their child's language strengths and difficulties should be considered for intervention planning (Jensen de López et al., 2021).

Furthermore, the effective implementation of pragmatic intervention programmes for preschool-age children with ASD and DLD requires a comprehensive understanding of the perceptions of key stakeholders, including parents and early childhood educators. It is essential to explore how these programmes are perceived and experienced by parents and other relevant stakeholders in real-world contexts for adapting interventions and ensuring their effectiveness and sustainability. Considering that parent satisfaction measures the degree to which parents view their expectations of children's needs are being met, it can provide a measure of quality care (Bairati et al., 2011), which will be important to the acceptance and translation of the intervention into clinical practice.

Some studies have analysed the parents’ perceptions about the effectiveness of language interventions (Baxendale et al., 2001; Stahmer et al., 2017), but they mainly refer to studies in which the intervention was coached by community providers or professionals and given by the parents themselves (parent-implemented interventions). Little attention has been paid to the subjective experiences and perceptions of those involved in the intervention but not delivering it.

In Baxendale et al.’s (2013) study, the perceptions of participation and outcomes of parents and teachers of eight children in the intervention arm of the Social Communication Intervention Project (SCIP) effectiveness study (Adams et al., 2012) were qualitatively analysed through semi-structured interviews. The results indicated that parents and teachers could associate intervention with perceived changes in the child. However, some changes were also attributed to factors other than SCIP intervention. They also described changes that were perceived by different people and that they considered to be the result of the intervention (Baxendale et al., 2013). However, it would have been interesting to analyse the perception of parents and teachers regarding the lasting improvement in social communication skills with this intervention.

Understanding parents’ and early childhood educators’ perceptions of an intervention programme is essential for informing the development, refinement, and dissemination of evidence-based interventions that should consider children's individual characteristics, needs, and preferences, as well as their families and educational providers. This study aims to analyse the perceptions of parents and early childhood educators following the implementation of the PICP (to children with ASD and children with DLD with pragmatic language difficulties).

## Materials and Methods

The data for this article were collected as part of a broad research project that aimed to analyse the effectiveness of the PICP with preschool-age children with ASD and children with DLD. A non-randomised controlled trial was carried out in early childhood educational settings. A full description of the trial methods and results can be consulted in Pereira et al. (2025b). Brief methodological details are provided in this section to give context to the data collected about parents’ and early childhood educators’ perspectives, which is the focus of this article. This study was approved by the Ethics Committee of the Health Sciences Research Unit: Nursing (734/12-2020). After receiving a detailed explanation of the study, written authorisation was obtained from the directors of the educational institutions. All parents signed a written informed consent authorising their child's participation. The children consent to their own participation verbally.

The intervention content was derived from the PICP but customised individually for each child. Collaborative goal setting between parents and early childhood educators was essential to tailoring and prioritising intervention goals according to each child's, parent’s, and educational provider’s needs. The baseline assessment included an evaluation of the parents’ and early childhood educators’ perspectives about the children's pragmatic language skills (EAC) (Seabra et al., 2021) and the application of an instrument (TL-ALPE) (Mendes et al., 2014) to assess general language ability (both secondary outcome measures). After the baseline assessment and before the intervention, a meeting was scheduled to discuss the children's pragmatic language difficulties and to jointly select three priority intervention goals across parents, early childhood educators, and the SLT. In cases where one of the members (parent or early childhood educator) could not attend the meeting, the content (strengths, needs, and priorities shared in the meeting) was discussed afterwards with those members (at the end of the day or the day after). The intervention goals were always set collaboratively, considering all the perspectives. An example of a goal, activity, materials, procedures, and strategies used was uploaded in supplementary material.

The children were allocated to an experimental (who received the intervention first) or a control group (waiting list)—the children in the control group did not receive the intervention sessions until the post-intervention assessment of the experimental group but received it after. All children received 24 PICP-based intervention sessions that were delivered at no cost, biweekly, for one hour, by an SLT (master, specialised in language and communication in children) with in-depth knowledge about the programme content and implementation and previous clinical expertise in providing intervention to children with pragmatic language difficulties in early childhood educational settings. The children did not receive a parallel intervention provided by another SLT during the study. All sessions were provided face-to-face in early childhood educational settings. Many communicative partners (e.g., peers with typical and atypical language development, early childhood educators) were involved in the activities to promote skills generalisation, positive relationships, and inclusion. After each session, feedback was provided to every parent and early childhood educator through photographic and videographic records (with prior written parental consent). After the intervention, that is, after the 24 sessions have been implemented in each group, quantitative methods were used to measure parents’ and early childhood educators’ perceptions through an adapted version of a satisfaction survey developed by Stahmer et al. (2017) (see section below).

### Participants

To be included in the study, all children had to have a diagnosis of DLD (children were diagnosed with DLD after a comprehensive language assessment with TL-ALPE (Mendes et al., 2014) applied by an SLT blind to the aims of the study; pragmatic competencies were evaluated with a parent/educator report—EAC (Seabra et al., 2021)) or ASD (clinical diagnosis provided by the neurodevelopmental pediatrician or psychiatrist according to DSM-V criteria, ADOS, and/or ADI-R). In addition, they had to be able to express themselves verbally, aged between 3;6 and 6;11 years, be native speakers of European Portuguese, and present at least two of five criteria that support the presence of pragmatic language difficulties (Pereira et al., 2025b).

All parents and early childhood educators whose child/student was enrolled in the PICP research project were asked to fill out a satisfaction survey after the implementation of the intervention by an SLT. Therefore, 72 participants among parents (*n* = 36) and early childhood educators (*n* = 36) were included in this study.

### Satisfaction Survey

In the study of Stahmer et al. (2017), the authors developed and used a satisfaction survey to analyse the parents’ perceptions of the content, design, and perceived effectiveness of a parent-implemented programme (Project ImPACT) for toddlers at risk for ASD. The survey included 19 statements about the project, and the parents scored each statement based on a seven-point Likert-type scale ranging from strongly disagree (1) to strongly agree (7) (Stahmer et al., 2017). An adapted version of this survey was used for the present study.

The first author analysed all the statements and selected the most appropriate considering the aims, concepts, and implementation of the PICP research project. This selection was done before the intervention started. Considering that the PICP was not implemented by parents or taught how to be implemented, statements such as: “*The parent coaching was clear, understandable, and helpful*”, “*The amount of training and support I received was sufficient for me to learn the intervention strategies*”, “*I use this intervention at home*”, “*The trainers were knowledgeable*”, “*I enjoyed this programme*”, “*I understand how to use the techniques at home during everyday activities*”, “*The homework assignments were clear and manageable*”, and “*I use the intervention with my child regularly*” (Stahmer et al., 2017) were not selected because they did not apply. Then, the fourth author revised the selected statements. No changes were made, and a total agreement was reached. The adapted survey version included 11 statements about the impact of the intervention. The questionnaire was administered in Portuguese, so the Portuguese version can be available from authors on request. As parents and early childhood educators filled out this survey, some modifications were made to make the statements suitable for both. For example, “*The intervention was effective in teaching my child's social communication skills*” was the statement used for parents, and “*The intervention was effective in teaching my students social communication skills*” was used for early childhood educators. Parents and educators should score each statement on a scale between 1 (totally disagree) and 7 (totally agree) according to their degree of agreement after 24 PICP-based intervention sessions in both groups. The scores were calculated firstly per statement but also by averaging the answers to all the statements to create an overall satisfaction rating for both raters.

### Data Analysis

The data collected was imported into Statistical Package for Social Sciences software (IBM SPSS Statistics, v29.0, IBM Corp., Armonk, NY, USA), and analysed considering descriptive statistics to characterise the parents’ and early childhood educators’ satisfaction. Means (*M*) and standard deviations (SD) were calculated per statement and globally, averaging the answers to all the statements to create an overall satisfaction rating for parents and early childhood educators.

## Results

### Participants

Thirty-six children were enrolled in a non-randomised controlled trial to explore the effectiveness of the PICP (Pereira et al., 2025b). The mean age (months) of the children in the control group was 54.2 ± 9.0, and in the experimental group was 53.9 ± 7.6. There were no statistically significant differences between groups in both categorical (condition, gender, and socioeconomic status) and continuous variables (age, EAC, and TL-ALPE scores) (see Table 1). All parents and early childhood educators whose children/students were involved in the PICP research project responded to the survey. According to the data collected, the parents’ mean age (years) after the intervention was 36.2 ± 7.1. The early childhood educators’ mean age (years) after the intervention was 54.1 ± 6.0. Thirty-five (97.2%) parents were mothers, and all the early childhood educators (100%; *n* = 36) were females.

**Table 1.** Sociodemographic and Clinical Characteristics of Children at Baseline.

	Control Group (*n* = 14)		Experimental Group (*n* = 22)		
Categorical variables	*n*	(%)	*n*	(%)	Statistical Results
Condition					
DLD	7	(50.0)	9	(40.9)	χ^2^(1) = 0.3; *p* = .734
ASD	7	(50.0)	13	(59.1)	
Gender					
Male	8	(57.1)	16	(72.7)	χ^2^(1) = 0.9; *p* = .471
Female	6	(42.9)	6	(27.3)	
SES					
High	3	(21.4)	2	(9.1)	Fisher = 1.9; *p* = .901
Medium-high	4	(28.6)	8	(36.4)	
Medium	1	(7.1)	2	(9.1)	
Medium-low	6	(42.9)	9	(40.9)	
Low	0	(0.0)	1	(4.5)	

Continuous variables	*M* ± SD		*M* ± SD		
Age (months)	54.2 ± 9.0		53.9 ± 7.6		*t*(34) = –0.1; *p* = .954
EAC-P	93.5 ± 20.7		91.0 ± 27.7		*t*(34) = –0.3; *p* = .774
EAC-E	82.3 ± 15.6		83.7 ± 22.3		*t*(34) = 0.2; *p* = .839
TL-ALPE	38.9 ± 21.6		41.0 ± 27.8		*t*(34) = 0.2; *p* = .808

DLD: developmental language disorder; ASD: autism spectrum disorder; SES: socioeconomic status; *M*: mean; SD: standard deviation; EAC-P: Escala de Avaliação de Competências Comunicativas rated by parents; EAC-E: Escala de Avaliação de Competências Comunicativas rated by early childhood educators; TL-ALPE: Teste de Linguagem - Avaliação da Linguagem Pré-Escolar.

Table 1 shows the sociodemographic and clinical characteristics of children at baseline. There were no statistically significant differences between groups in both categorical and continuous variables.

### Satisfaction Survey

The overall satisfaction survey results scored by parents and ECEs were positive, with an average of 6.83 ± 0.29 for parents and 6.60 ± 0.49 for ECEs, in a total of 7. Table 2 shows the results of the satisfaction survey, calculated per statement but also globally, considering parents’ and early childhood educators’ ratings. In Table 3, the results are specified by group. There were no statistically significant differences (*p* > .05) between conditions (ASD/DLD) and groups (experimental/control) in parents’ or educators’ ratings.

**Table 2.** Satisfaction Survey Results.

Question	Parents		Early Childhood Educators	
	*M* ± SD	Range	*M* ± SD	Range
1. This is an acceptable intervention for my child/student's social communication skills	6.83 ± 0.70	4–7	6.81 ± 0.47	5–7
2. I would suggest this intervention to other parents	6.94 ± 0.23	6–7	6.81 ± 0.58	4–7
3. The goals of the intervention are important to my child's/student's functioning at home/school	6.83 ± 0.56	4–7	6.78 ± 0.48	5–7
4. This intervention was a good way to teach social communication skills to my child/student	6.97 ± 0.17	6–7	6.83 ± 0.45	5–7
5. I understand which skills my child/student was working on and why	6.92 ± 0.28	6–7	6.86 ± 0.42	5–7
6. The intervention was effective in teaching my child/student social communication skills	6.89 ± 0.32	6–7	6.75 ± 0.50	5–7
7. I feel that my child/student improved his/her social engagement as a result of this programme	6.86 ± 0.42	5–7	6.53 ± 0.74	4–7
8. The intervention will produce lasting improvements in my child/student's social communication skills	6.86 ± 0.42	5–7	6.33 ± 0.79	4–7
9. The intervention quickly improved my child/student social communication skills	6.69 ± 0.58	5–7	6.22 ± 0.83	4–7
10. Other behaviours related to social communication were also improved by the intervention	6.69 ± 0.75	4–7	6.31 ± 0.82	4–7
11. Using the intervention not only improved my child/student's social communication skills at home/school but also in other settings (e.g., community)	6.61 ± 0.87	4–7	6.42 ± 0.97	4–7
Overall	6.83 ± 0.29	4–7	6.60 ± 0.49	4–7

*M*: mean; SD: standard deviation.

Adapted from Stahmer et al. (2017).

**Table 3.** Satisfaction Survey Results Specified by Group (Experimental/Control).

	Parents		Early Childhood Educators	
	Experimental	Control	Experimental	Control
Question	*M* ± SD	*M* ± SD	*M* ± SD	*M* ± SD
1. This is an acceptable intervention for my child/student social communication skills	6.86 ± 0.64	6.79 ± 0.80	6.82 ± 0.50	6.79 ± 0.43
2. I would suggest this intervention to other parents	6.95 ± 0.21	6.93 ± 0.27	6.77 ± 0.69	6.86 ± 0.36
3. The goals of the intervention are important to my child's/student's functioning at home/school	6.73 ± 0.70	7.00 ± 0.00	6.82 ± 0.50	6.71 ± 0.47
4. This intervention was a good way to teach social communication skills to my child/student	6.95 ± 0.21	7.00 ± 0.00	6.86 ± 0.47	6.79 ± 0.43
5. I understand which skills my child/student was working on and why	6.86 ± 0.35	7.00 ± 0.00	6.91 ± 0.43	6.79 ± 0.43
6. The intervention was effective in teaching my child/student social communication skills	6.91 ± 0.29	6.86 ± 0.36	6.82 ± 0.50	6.64 ± 0.50
7. I feel that my child/student improved his/her social engagement as a result of this programme	6.86 ± 0.47	6.86 ± 0.36	6.59 ± 0.73	6.43 ± 0.76
8. The intervention will produce lasting improvements in my child/student's social communication skills	6.77 ± 0.5	7.00 ± 0.00	6.36 ± 0.85	6.29 ± 0.73
9. The intervention quickly improved my child/student's social communication skills	6.64 ± 0.66	6.79 ± 0.43	6.18 ± 1.01	6.29 ± 0.47
10. Other behaviours related to social communication were also improved by the intervention	6.59 ± 0.91	6.86 ± 0.36	6.36 ± 0.90	6.21 ± 0.70
11. Using the intervention not only improved my child/student's social communication skills at home/school but also in other settings (e.g., community)	6.55 ± 0.91	6.71 ± 0.83	6.55 ± 0.96	6.21 ± 0.98
Overall	6.79 ± 0.30	6.88 ± 0.29	6.64 ± 0.56	6.51 ± 0.34

M: mean; SD: standard deviation.

Adapted from Stahmer et al. (2017).

## Discussion

This study aimed to analyse the perceptions of parents and early childhood educators following the implementation of the PICP to preschool-age children with ASD and children with DLD. The results indicate that parents and early childhood educators had positive perceptions about the acceptability and effectiveness of the programme, supporting the successful implementation of the PICP with preschool-age children with pragmatic language difficulties arising from ASD or DLD. These results enhance the previous research evidence on the effectiveness of the PICP, corroborating the outcome measures results that revealed a statistically significant improvement in language competencies after intervention (Pereira et al., 2025b).

Although the mean scores of both parents and early childhood educators across statements were high, the parents’ scores were always slightly higher, which is also reflected in their overall satisfaction. This is consistent with the mean total scores and perceived pragmatic language skills in the secondary outcome measure (EAC) rated by parents and early childhood educators, who were also higher when rated by parents (Pereira et al., 2025b). The literature emphasises that parents and teachers shared concerns about language difficulties but often had different perspectives about the impacts of impaired social communication (Baxendale et al., 2013).

The ratings on the survey statements one (*This is an acceptable intervention for my child/student's social communication skills*) and two (*I would suggest this intervention to other parents*) were high from parents and early childhood educators, indicating strong acceptability (deeper engagement around the concept of acceptability is needed and to deem whether or not is acceptable) and support for the intervention. Also, statements related to the impressions of the intervention, namely three (*The goals of the intervention are important to my child's/student's functioning at home/school*) and four (*This intervention was a good way to teach social communication skills to my child/student*), were highly scored by both parents and early childhood educators, which highlights the symbiosis between the content of the intervention and the real needs of the children. Considering intervention planning, parents and early childhood educators were involved in goal selection for each child and were aware of the activities carried out throughout the project. Therefore, they indicated that they understood which skills the child/student was working on and why, scoring statement five very high. Similar results were found in the study by Stahmer et al. (2017) following a parent-implemented intervention, although the mean scores in the present study were relatively high. This emphasises that even when the intervention is implemented by an SLT, the parents and early childhood educators can be deeply involved in the intervention process.

Regarding perceived effectiveness, parents and teachers strongly indicated that *the intervention was effective in teaching their children/students social communication skills and* that *the children/students improved his/her social engagement as a result of the intervention*. In Stahmer et al.’s (2017) study, parents also considered the intervention effective. Moreover, in the present study, parents believed that *the intervention would produce lasting improvements in their children*; the *intervention quickly improved his/her children's social communication skills,* and they considered that *other behaviours were also improved by the intervention*. A strong belief was also verified considering the generalisation of the skills learned and improved during the intervention to other settings. Similar results were found in the study by Stahmer et al. (2017), although they were slightly lower when compared to the present study. Furthermore, when asked about whether *the intervention quickly improved his/her children's social communication skills, other behaviours were also improved by the intervention*, and whether *the intervention has improved her/his child's social communication skills at home but also in other settings*, the results were also more moderate (Stahmer et al., 2017). Considering the early childhood educators’ perceptions in the present study, although there were also high scores on all the statements, the scores were more moderate from statements eight to 11 (mean scores between 6.22 ± 0.83 and 6.42 ± 0.97) compared to the other statements and the parents’ scores (above 6.5). There seems to be some moderation regarding quick and lasting improvements in social communication skills, setting generalisation and improving other behaviours as a result of the intervention. This is an extremely important criterion considering the effectiveness of an intervention, although not always studied (Baxendale et al., 2013).

## Limitations

Several limitations should be acknowledged. Although parents and early childhood educators globally believe that the improvements in social communication (achieved as a result of the intervention) will last, it would have been essential to conduct a blind evaluation a few months after the end of the intervention (follow-up assessment) to support this. The perceptions of the child participants and the communicative partners involved in the intervention session would also have provided important additional insight into the intervention and, therefore, should be considered in future studies. Open statements should also be included so that different stakeholders can suggest improvements in the intervention.

Considering that all statements are formulated positively, this could have also impacted the results. Also, the authors did not statistically analyse whether the statements were equally easy or difficult, did not guarantee that the items were understood as intended by parents and educators and did not analyse the scale dimensionalities. These gaps limit the ability to draw conclusions based on average scores across items. Since there was constant communication with parents and educators, they probably learned more about pragmatic language during the intervention, which might have influenced their scoring. Furthermore, considering that the intelligence quotient may impact pragmatic language and the children's response to intervention, it should have been evaluated.

## Conclusions

Exploring perceptions of an intervention's value is essential in translating experimental treatments into clinical practice. The overall perceptions of parents and educators support the successful implementation of the PICP with preschool-age children with pragmatic language difficulties and enhance the previous research evidence on its effectiveness with children with ASD and DLD (Pereira et al., 2025b).

Furthermore, it highlights the importance of practice based on research evidence and clinical expertise, considering the values and perspectives of the caregivers. Future effectiveness studies should consider the perceptions of parents and other relevant stakeholders beyond the long-term effects of the intervention measure through instruments directly applied to the child. Also, future studies should consider the psychometric properties of the instruments to assess satisfaction.

## Implications

The results of the present study, along with the previously published significant, and clinical improvements in language competencies following PICP-based intervention (Pereira et al., 2025b), support the PICP’s effectiveness in improving language competencies in preschool-age children with ASD and DLD through stakeholders’ perceptions. Clinicians and policymakers can now implement and recommend an evidence-based intervention for preschool-age children with ASD or DLD and pragmatic language difficulties.

## Supplemental Material

sj-docx-1-dli-10.1177_23969415251330465 - Supplemental material for Parents’ and Early Childhood Educators’ Perceptions after the Implementation of the Pragmatic Intervention Programme (PICP) Supplemental material, sj-docx-1-dli-10.1177_23969415251330465 for Parents’ and Early Childhood Educators’ Perceptions after the Implementation of the Pragmatic Intervention Programme (PICP) by Tatiana Pereira, Ana Margarida Ramalho and Marisa Lousada in Autism & Developmental Language Impairments

## Notes

1. Pragmatic language difficulties refer to challenges in using language effectively and appropriately in social contexts, including understanding conversational rules, interpreting non-literal meanings, and maintaining coherent interactions. These difficulties may be present in several disorders with different severity levels (e.g., ASD and DLD; Cummings, 2021).
