# Supplementary material for: Parents’ and Early Childhood Educators’ Perceptions after the Implementation of the Pragmatic Intervention Programme (PICP)
Source: Autism Dev Lang Impair. 2025 May 12;10:23969415251330465. doi: 10.1177/23969415251330465 (PMC12075966; doi:10.1177/23969415251330465)
Supplement: sj-docx-1-dli-10.1177_23969415251330465 - Supplemental material for Parents’ and Early Childhood Educators’ Perceptions after the Implementation of the Pragmatic Intervention Programme (PICP) [file sj-docx-1-dli-10.1177_23969415251330465.docx]

Supplementary Material

Table 1. Example of a goal, activity, materials, procedures and strategies used.

| Goal: The child should be able to recognize emotions such as joy, sadness, fear, surprise, disgust and anger, through facial expression. |
| --- |
| Activity: Observe and recognise! |
| Materials: Images of emotions |
| Procedures and Strategies:  To carry out the proposed activity, the SLT should use a set of images that illustrate various emotions. After exploring each of the emotions with the child, the SLT should arrange the images on a surface (using a number of stimuli appropriate to each child). It is intended that, in the first instance, the child identifies the emotion present in each image. Subsequently, the SLT should ask the child to name the emotion illustrated. The SLT can also simulate the facial expression associated with each emotion, encouraging the child to name and recognise the emotion in a real expression. The participation of other communicative partners (adults/children) is suggested. |
